# Reduced growth and proliferation dynamics of nasal epithelial stem/progenitor cells in nasal polyps *in vitro*

**DOI:** 10.1038/srep04619

**Published:** 2014-04-09

**Authors:** Xue Min Yu, Chun Wei Li, Siew Shuen Chao, Ying Ying Li, Yan Yan, Xue Ning Zhao, Feng Gang Yu, Jing Liu, Liang Shen, Xin Liang Pan, Li Shi, De Yun Wang

**Affiliations:** 1 Department of Otolaryngology, Qilu Hospital, Shandong University, China; 2 Department of Otolaryngology, National University of Singapore, National University Health System, Singapore; 3 Department of General Health Care II, Shandong Provincial Hospital affiliated to Shandong University, China; 4 Biostatistics Unit, National University of Singapore, National University Health System, Singapore; 5 These authors contributed equally to this work.

## Abstract

Basal cells in nasal epithelium have stemness/progenitor characters and play essential roles in the epithelial remodeling in nasal polyps (NP). We investigate whether the human nasal epithelial stem/progenitor cells (hNESPCs) from patients with NP are inherently distinct from those obtained from healthy controls. Epithelial basal cells were isolated and cultured for four passages from NP tissues and control nasal mucosa. hNESPCs from controls were stained positively with stem cell marker p63 and KRT5 and presented a consistent high Ki67 expression level over four passages. In contrast, hNESPCs from NP patients showed: i). a reduced growth and proliferation rate at each passage by evaluating colony-forming efficiency and doubling time; ii). a lower percentage of Ki67^+^ cells among p63^+^ cells in the colonies in late passages, which was also confirmed by immunostaining in the NP tissues. Thus reduced growth/proliferation dynamics in hNESPCs from NP could be an important pathological phenomenon in NP development.

Nasal polyps (NP) is characterized by increased inflammatory cell infiltration and abnormal tissue remodeling^1^. Emerging evidence has demonstrated that epithelium from NP patients plays an important role in the pathogenesis of NP. In patients with NP, the epithelium is attacked by various stimulants, leading to acute or chronic injury and dysregulated restitution followed by aberrant remodeling^2^. Our previous studies reported a down-regulation of activator protein 1 (AP1) and its related genes (e.g., COX2, IL6, and epidermal growth factors) was associated with the damage of epithelial structure^3^; while up-regulation of p63 in basal cells was implicated in the epithelial hyperplasia in NP^4^. In addition, alterations of tight junction proteins^5^, cell-cell adhesion molecules^6^,^7^ and Toll like receptors^8^,^9^ may contribute to the defect of the epithelial barrier and host defense function in NP mucosa. *In vitro* studies also showed that the inhibitor (CP110) of ciliogenesis increased in the epithelial differentiated cells derived from NP tissues, resulting in poor ciliation^10^. Collectively, these data suggest that the biological properties and functions of NP epithelium are dysregulated.

There are four major cell types in healthy nasal epithelium, including basal cells, ciliated cells, non-ciliated columnar cells and goblet cells^11^. Basal cells are considered to have stemness and progenitor properties, which can self-renew and differentiate into other epithelial cell types^12^,^13^. In our recent study, we have successfully isolated and cultured human nasal epithelial stem/progenitor cells (hNESPCs) from human inferior turbinate tissues in a serum-free culture method^14^. This technical advance facilitates studies on the pathological mechanisms underlying abnormal epithelial repair and remodeling in inflammatory airway diseases, such as NP.

The most reported studies are investigations of the pathological changes in epithelium, together with the underlying molecular markers and gene regulations in NP mucosa tissues, but no study has investigated the biological properties of hNESPC *in vitro*. The aim of this study was based on the hypothesis that there are intrinsic phenotypic differences between NP and healthy nasal epithelium. Therefore, in this study, we investigated growth and proliferation properties of hNESPCs in an *in vitro* cell culture system and further confirmation was performed in nasal mucosal tissue obtained from healthy subjects and NP patients.

## Results

### Growth dynamics of hNESPCs from patients with NP and healthy controls

**Figure 1:**
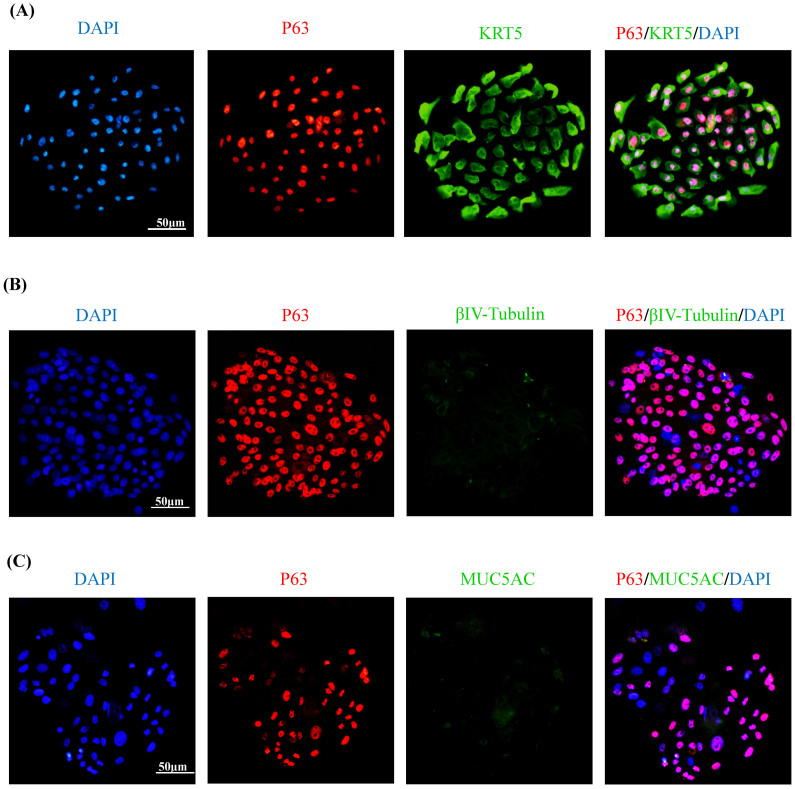
Charaterization of the cells in colonies by using immunofluorescence assay. Representative pictures show the p63/KRT5 double-positive cells in P1 cells (A). BetaIV-tubulin or MUC5AC staining is negative in the hNESPC cultures (B & C). Original magnification 200× (scale bar = 50 μm).

The cell cultures reached confluence at about 6 days and demonstrated a typical cobblestone shape of epithelial basal cells, which were successfully maintained up to four passages. More than 90% of the cells in the colonies were p63 positive, and among these cells, approximately 90% were co-localized with KRT5; while they did not express any differentiated nasal epithelial cell markers (e.g., betaIV-tubulin and MUC5AC) (Figure 1). Another common stem cell marker KRT14 was also stained in the colonies, but only a subset of p63 or Ki67 positive cells expressed KRT14 (Supplementary Fig. S1A &1B).

**Figure 2:**
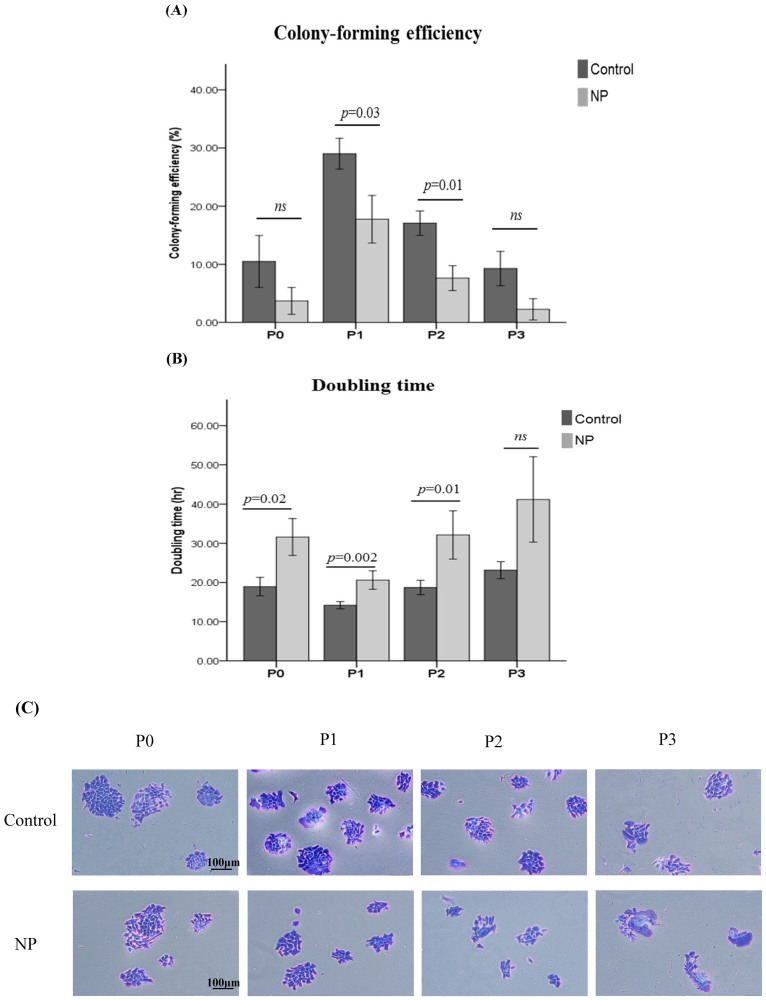
Comparisons of CFE and doubling time at each passage (P0 to P3) of the hNESPCs from NP versus healthy controls. The value of CFE was given as a percentage (A) and the values of doubling time (hour) are shown in panel (B). Clustered column charts are shown with mean plus 95% confident interval (CI). Statistical analyses were performed with the linear mixed models. *P* values were considered significant at less than 0.05. Representative staining pictures of colonies over subsequent passages in cultures from NP and controls (C). Original magnification 100× (scale bar = 100 μm).

To study the growth rates of hNESPCs over passages, it is required to observe the exponential phase of cell proliferation within the passage. Our previous study showed that, for each passage, the cells seeded in the first 3 days instead of the day of confluence reflected the best capacity for cell proliferation and were also easy to observe under the microscope^14^. Initially, P0 culture may contain both progenitor cells, and other cell types (e.g., leukocytes, ciliated cells, and goblet cells) which cannot adhere on the culture plate. After 2 days, these cells were removed by changing the medium. Although a small amount of fibroblasts existed in the early stage of P0, they could not survive in the serum-free culture medium. Therefore, hNESPC can be considered the most dominant adherent cell type in the colonies of P0 culture. hNESPCs from both NP and control tissues showed a similar growth pattern throughout the 4 passages (Figure 2A & 2B): 1). P0 culture showed a little bit slower growth rate as compared to P1; 2).The cell cultures from P1 demonstrated the highest colony forming capacity and the CFE values decreased at passage 2 and 3; 3).in all samples there was a marked increase in cell doubling time from P1 to P3 over repeated passages. The age effect on the measurement of cell grow dynamic was also analyzed in all NP and control subjects. The results showed that there was no significant correlation between age and the values of CFE/doubling time (Supplementary Table S1).

Comparison between hNESPCs from NP and control tissues based on their morphology was also performed through serial passages. hNESPCs showed a similar morphologic character in colonies from NP and control cell cultures in P0 and P1. However, more “fried-egg” shape phenotype cells were seen from the cell cultures of NP tissues in both P2 and P3 than those from controls (Figure 2C and Supplementary Fig. S2A). These cells were almost all β-galactosidase positive, showing a light blue staining mostly in perinuclear region, indicating more senescent cells in the colonies (Supplementary Fig. S2B). Although the CFE and doubling time were changed in cells from controls, the morphology pattern of hNESPCs in colonies remained consistent over continual passages (Figure 2C).

We further compared the growth and proliferation rate of hNESPC cultures isolated from NP and healthy controls. Cells from control tissues proliferated more rapidly than those from NP tissues over subsequent passages, showing higher CFE values (difference from 11.3% to 8.5% through P1 to P3) and shorter doubling times (difference from 6.5 hr to 22 hr through P1 to P3). However, a significant difference in these two measurements existed mainly in cells from P1 and P2 (Figure 2A & 2B). In addition, the variations (based on standard deviation) of CFE and doubling time values were larger in cell cultures derived from NP tissues.

### Assessment of protein expression of p63 and ki67

**Figure 3:**
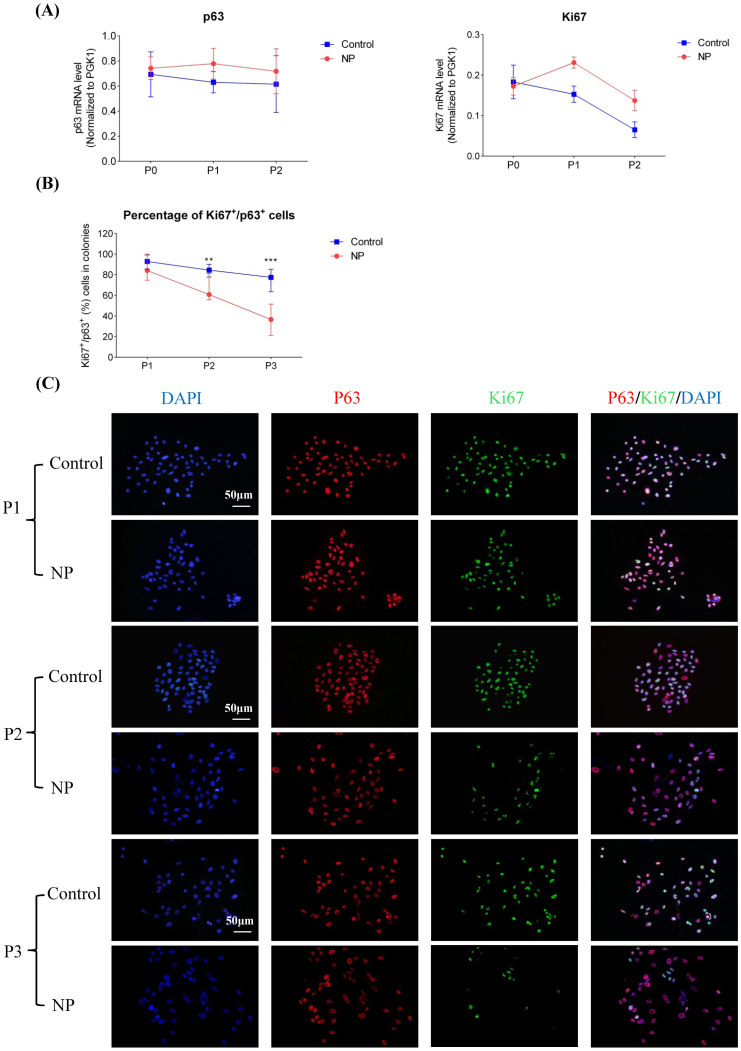
Comparisons mRNA/protein levels of p63 and Ki67 at different passages (P0 to P3) of the hNESPCs from NP versus healthy controls. p63 and Ki67 mRNA levels were determined by means of real-time RT PCR (panel A). All target mRNA relative expression levels were given as ratios of PGK1 transcript levels. Line charts are shown with mean ± SE. p63 and Ki67 protein levels were determined by means of immunofluorescence assay (B). Mann-Whitney test was used to analyze the difference of Ki67^+^/p63^+^ cell percentage in colonies between NPs and controls at each passage. Symbol “**” means the p-value is less than 0.01, while symbol “***” means the p-value is less than 0.001. Representative pictures show the p63/or Ki67 single-positive cells and p63/Ki67 double-positive cells (panel C). Original magnification 200×. (scale bar = 50 μm).

The immunocytochemistry results showed that all cultures were stained positive for p63 throughout the four passages and there was no obvious difference of p63 expression between cells from NP and those from healthy controls (Figure 3C). In hNESPCs from controls, Ki67 protein expression continued to be intensively stained and the percentage of Ki67^+^ cells among p63^+^ cells in the colonies remained steady over continual passages (median value, range from 77% to 93% through P1 to P3) (Figure 3B & 3C). However, in hNESPCs from NP samples, the ratio of Ki67^+^/p63^+^ cells was significantly lower in P2 and P3 as compared to those cells from controls and the percentage of Ki67^+^ over p63^+^ cells decreased from P1 to P3 (median value, 84%, 61%, and 36% respectively) (Figure 3B & 3C).

### Assessment of mRNA expression of p63 and ki67

Expression of p63 mRNA was almost constant from P0 to P1 in both NP and control cell cultures. The difference of p63 mRNA levels was not significant between cells of NP and controls from P0 to P2 (Figure 3A). The ki67 expression level decreased from P1 to P2 in cells from NP and control samples. In addition, ki67 mRNA level was lower in hNESPCs from NP as compared to controls in P1 (1.5-fold, a borderline significant trend, *p* = 0.05) and P2 (2.1-fold, approaching borderline of significance, *p* = 0.07) (Figure 3A). No RNA analysis was made in P3 due to the small number of cells collected in P3.

### Evaluation of staining patterns of p63 and Ki67 in nasal tissues

**Figure 4:**
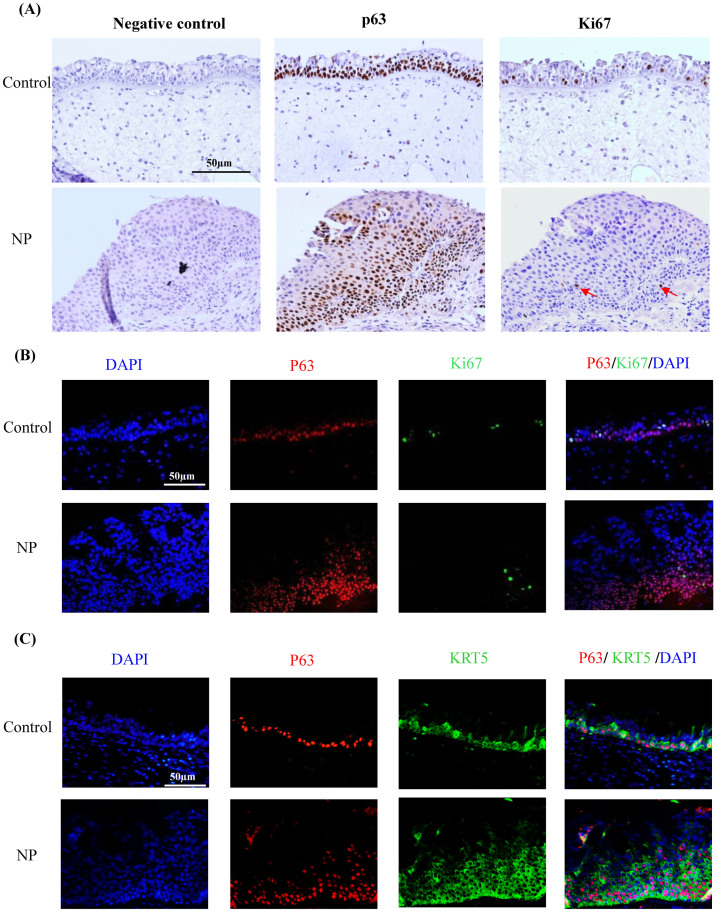
Expression of p63 and Ki67 proteins determined by means of immunohistochemistry (panel A) and immunofluorescence (panel B) in the nasal mucosa of representative healthy control subjects and patients with NP. Doubling staining of p63 and KRT5 is shown in (panel C). Original magnification 200×. (scale bar = 50 μm).

To explore the proliferation status of nasal epithelial basal cells *in vivo*, we further analyzed expression levels of p63 and Ki67 in nasal mucosa from the same NP and control subjects. p63 immunostaining was confined to the basal cells and p63 positive cells increased in NP epithelium when compared with those in healthy controls (Figure 4). Staining for Ki67 was also mostly restricted to the basal cells in control epithelium. Interestingly, Ki67 immunostaining decreased in the hyperplasia or metaplasia areas of NP epithelium as compared to control epithelium (Figure 4). Similarly, the percentage (median, 25^th^–75^th^ percentile) of Ki67^+^ cells among p63^+^ cells was significantly lower (*p* < 0.001) in the hyperplasic NP epithelium (7.3%, 4.3%–9.9%) than in healthy controls (13.1%, 11.4%–17.3%) by immunofluorescence staining (Supplementary Fig. S3). In addition, the colocalization of p63 and KRT5 was also confirmed in nasal tissue staining (Figure 4). However, no KRT14 expression was found in those p63 positive cells from healthy control (Supplementary Fig. S1C); while only weak and sparse staining of KRT14 was found in p63 positive cells in the remodeled epithelium from NP patients (Supplementary Fig. S1D).

## Discussion

Although Th2-skewed eosinophilic dominated inflammation is the important pathological feature in NP, emerging evidence suggests that nasal epithelium plays an active role in the immune pathological changes, dysregulation of host defense and tissue remodeling in NP. It is not yet possible to distinguish between cause and effect during epithelium remodeling, nor are there clear roles for the many factors involved in respiratory infections and inflammatory diseases, due to a lack of critical information about epithelial cell responses. Most reported data are from lower airway studies or animal models. Therefore, research based on hNESPCs can help illuminate the pathophysiology of nasal airway disease from a different, more specific perspective.

Basal cells are considered to be the adult stem cells in the airway that play a critical role in epithelial repair^12^,^13^. To date, most *in vitro* studies on airway epithelial cells used an air liquid interface (ALI) culture which contains mainly differentiated ciliated cells and goblet cells, but less epithelial basal cells^10^. To our knowledge, this is the first study showing the growth properties of the hNESPCs derived from NP tissues versus those from healthy controls *in vitro*. We have successfully compared these two different originated hNESPCs over four subsequent passages and found that the cells isolated from NP epithelium exhibited lower growth and proliferative dynamics than the healthy controls. In addition, the distributions of CFE and doubling time values were more dispersed from P1 to P3 in NP hNESPCs, implying the heterogeneity of growth patterns in NP isolated cells.

We also found that the expression level of basal cell markers p63 as well as KRT5 were stable in colonies cultured from NP and control epithelium throughout all passages, indicating the stem/progenitor epithelial cell linage. Ki67 is a proliferation marker and is considered to drive cell cycle progress with a peak expression during mitosis^15^. Even the cells in colonies consistently expressed p63, while there was a decreased proportion of Ki67^+^ cells in p63^+^ cells which was significant in NP epithelium, but it remained consistent in the colonies from the healthy controls. In addition, there were more senescent cells found in P2 and P3 cultures from NP samples compared to those from controls. Similar results were also obtained from quantitative PCR assays, showing a stable p63 mRNA level but with a decreased Ki67 level through P0 to P1 in all cell cultures, and Ki67 expression was further lower in cells derived from NP samples as compared to the controls. These results were concordant with the observation of CFE and doubling time in cell cultures, supporting the evidence of reduced growth and proliferation potential in hNESPCs derived from NP epithelium.

Dysregulation of airway epithelial basal cells can cause the aberrant remodeling process (e.g., basal cell hyperplasia, goblet cell hyperplasia, and squamous metaplasia)^2^,^13^. Our previous study found that p63 expression levels were higher in epithelium from patients with NP than in healthy controls, and it was associated with the severity of epithelial hyperplasia^4^. However, it is not known if inflammatory hyperplasia occurs commonly when there is an increase in a poorly differentiated cell layer that may stain for basal markers and does not form a proper epidermal barrier. Cohen et al reported that ciliary maturation was reduced in epithelial differentiated cells from chronic rhinosinusitis (CRS) epithelium and there was an increase in the negative regulator of ciliogenesis (CP110) in CRS cell cultures which may result in poor ciliation^10^. In addition, our tissue staining results of p63 and Ki67 showed a lower percentage of Ki67^+^ cells among p63^+^ cells in the hyperplasia/metaplasia area of NP epithelium as compared to healthy mucosa, implying a limited extent of epithelial restitution in NP. Our results are in line with the findings of decreased expression of proliferation markers (Ki67) in the epithelium of asthmatic children^16^. However, the p63 staining extent was similar in the cells from NP and control samples. This discrepancy in p63 staining pattern between tissues and cell cultures is mainly attributed to the different microenvironment in *in vivo* and *in vitro* condition, as there may be crosstalk between epithelial basal cells and other sub-epithelial cells (e.g., fibroblasts and leukocytes) via a variety of signaling pathways in nasal mucosa. Nonetheless, all these *in vitro* and *in vivo* data suggest that although the basal cells are p63 positive, it might not mean such cells could perform a normal physiological function (e.g., cell differentiation) or respond properly to the damage (e.g., epithelial repairing) in chronic inflammatory tissues, indicating that the nasal epithelial basal or stem/progenitor cells from NP mucosa are intrinsically abnormal.

There are some limitations in this *in vitro* study of hNESPCs. The *in vitro* findings are not able to represent a whole picture of the *in vivo* environment as the effects of those epithelial differentiated cells and other infiltrated cells cannot be investigated in the culture system. Since most epithelial stem/progenitor cells rapidly lose their-multiple differentiation potential during *in vitro* expansion^17^, the cells cannot be passaged multiple times. We found that the trend of growth dynamic was reduced gradually following P1 to P3 in the cells from either NP or control tissues, but this phenomenon did not start on P0, as the growth and proliferation rate of P0 was lower than P1. This could be due to the heterogeneity of cells in early culture period (within the first 2 days) of P0, where they were cultured from primary cells which were directly isolated from nasal tissues. But it did not change our major conclusion, because the staining results of basal cell markers (p63 & KRT5) confirmed that hNESPC was the most predominant adherent cell type in the colonies of P0 culture, and the hNESPC in P0 still grow faster in control-derived cells than NP-derived cells. Nevertheless, the influence of those other cell populations on P0 cell is not clear, and it is recommended that the basal cells could be sorted before starting the culture. Finally, although there is some evidence to show that there are intrinsic differences between hNESPCs in NP and control tissues, the molecular mechanism underlying this change needs to be further clarified in the future, and more gene markers need to be identified.

In conclusion, our study demonstrated significantly reduced growth and proliferation dynamics in hNESPCs from NP epithelium. These intrinsic differences in growth and proliferation properties could be the main cause of the persistence and aberrant remodeling in NPs.

## Methods

### Human nasal samples

Four adult patients with NP (age, mean ± SD, 50 ± 3) 4 healthy control subjects (age, mean ± SD, 40 ± 10) without NP were recruited from the National University Hospital of Singapore. All NP patients were bilateral with Grade-3 NP^1^ which had completely obstructed the nasal cavity. Biopsies were obtained during functional endoscopic sinus surgery. Biopsies of inferior turbinate (IT) mucosa were obtained from non-NP patients with septal deviation, who had been scheduled for nasal septoplasty surgery. This tissue served as a healthy control. All control subjects did not have allergic symptoms and sinusitis. None of the NP patients and controls had a concurrent upper respiratory infection, asthma, or other systemic diseases. In addition, the patients had not used any form of glucocorticosteroids or antibiotics within three months before the study. The methods and experiments were carried out in strict accordance with the approved guidelines and regulations. Approval for this study was obtained from the National Healthcare Group Domain- Specific Review Board of Singapore (Singapore). All participants were given written informed consent in this study. The consent procedure was approved by the ethics committees from the National Healthcare Group Domain- Specific Review Board of Singapore (Singapore).

### hNESPC cultures

Fresh specimens were immediately washed with cold PBS and were digested by Dispase II at 4°C overnight (Sigma, St. Louis, MO). A single-cell suspension was obtained and made ready for culture. NIH 3T3 cells were seeded at 2 × 10^4^/cm^2^ in 24-well plate and were treated with 10 μg/ml of mitomycin C (Sigma) to arrest growth at 37°C for 2.5 hours. Primary cells (P0) were seeded on the pretreated NIH 3T3 cells (ATCC, Manassas, VA) at optimized 2 × 10^3^ cells/cm^2^ in 24-well plates and were cultured with in-house serum-free medium. Other seeding densities were tested in the beginning of the experiment. By seeding a serial number of 200, 1,000, 2,000, 4,000 and 10,000 cells in each well of 24-well plate, we found that: 1). No clones could be formed in the cultures from passage 0 to 3 when seeding 200 or 1,000 cells initially; 2). For 2,000 cells/well, clones could be observed but were self-differentiated; 3). For 10,000 cells/well, the clones were formed too early to be observed in the initial day and were easily merged to form large clones. Therefore, 4000 cells per well was the optimal cell density for both cell growth and evaluation of colony forming efficiency and doubling time of hNESPCs from P0 to P3, Only epithelial basal cells, but no other cell types such as epithelial differentiated cells, fibroblasts or leukocytes can grow and proliferate in this culture system. When the cells were more than 80% confluent, cells were enzymatically digested and single-cell suspensions were subcultured at the same cell density as primary cell cultures. The hNESPC was cultured for 4 passages (i.e., P0, P1, P2, and P3) in the current study. The details of the culture protocols have been described previously^14^.

### Evaluation of doubling time,colony-forming efficiency and cell proliferation

**Figure 5:**
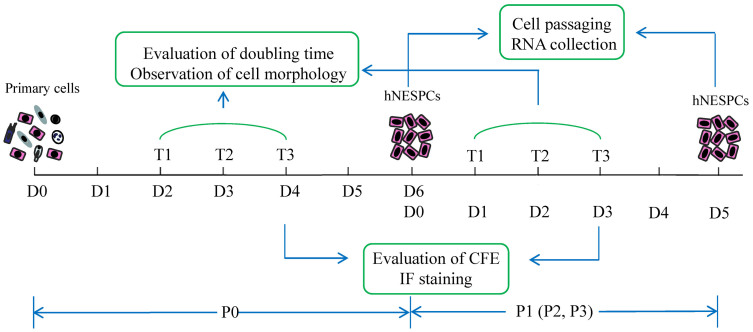
Flow chart of the study showing the experimental design. Primary cells from nasal tissues were isolated and seeded on 3T3 feeders on Day 0 in passage 0 (P0). The culture conditions favor human nasal epithelial stem/progenitor cells (hNESPCs), therefore, other cell types like fibroblasts, lymphocytes, and epithelial differentiated cells could not grow in this system. After 48 hours, 10 random colonies were marked and tracked for 3 days. Observation of the cell morphology and evaluation of doubling time were performed on T1, T2, and T3. On T3, the cells in three wells of the 24-well plate were fixed and stained for calculation of CFE and immunofluorescence assay. On the day of cell confluence (Day 6), the hNESPCs in P0 were enzymatically digested and subcultured. Cellular RNA was also obtained at this point. Hereafter, the cells were continually passaged to P3. The same observation and evaluation procedures were also done in P1, P2, and P3.

The experimental design including observation time points, sample collection, and evaluation methods is described in Figure 5. From P0 to P3, cell clusters started to form within 1 to 2 days after plating. The cell clusters (about 4 cells) were randomly chosen, marked and tracked for 72 hours (hrs.) (Supplementary Fig. S4). Cell numbers within each colony were recorded at three different time points (every 24 hrs.) for the calculation of doubling time using the following formula: Td = Δt × Lg2/(LgNt − LgN0). Td: doubling time; Δt: duration of culture; N0 = the cell number of single colony; Nt = the cell number after t hrs. culture^18^. Ten colonies for each sample in every passage were randomly chosen. At 72 hrs, cells in 24 well plates were fixed for 15 min with 4% paraformaldehyde (PFA) and then stained with Brilliant Blue (Sigma) for easy visualizing (Supplementary Fig. S4). Images of colonies were captured at 100× magnification. Those cell clusters with more than 8 cells were considered as a colony, which was assumed to be derived from one cell. Total colonies were counted in triplicate wells per sample per passage and used for the calculation of colony-forming efficiency (CFE) which was calculated by (number of colonies/number of cells seeded) × 100%.

Cell proliferation assay using CyQUANT® kit (Life Technologies) was also performed following the manufacture's protocol in the initial stage of this study. It can be used to determine the cell number by evaluating the fluorescence signals when the CyQUANT® GR dye bind to cellular nucleic acids. In the Days 1, 2, 3, and 4 (as shown in Figure 5), hNESPCs were detached by Acutase (Life Technologies), washed by PBS, and the cell pellets were frozen at −70°C until use (for up to four weeks). When the samples were ready, CyQUANT® GR dye/cell-lysis buffer was added to each frozen cells and incubated for 5 minutes at room temperature protected from light. The reaction mixtures were transferred to 96-well plate and fluorescence intensity was measured by Synergy™ H1 microplate reader (BioTek®, Winooski, Vermont) for excitation at about 480 nm and emission at about 520 nm. A cell number standard curve (using the same hNSPECs) was created in parallel with the experiment for converting sample fluorescence values into cell numbers. The results showed the cell number was increased following the culture period (Supplementary Fig. S5). In addition, the growth dynamic (from P0 to P3) of NP-derived cells was slower than that of control cells (Supplementary Fig. S5), which were comparable to the trend measured by another two assays (CFE and doubling time). Because CFE and doubling time assays can monitor the cell proliferation dynamic in a clone level, and the CyQuant assays required more cells for the experiment, we only chose the CFE and doubling time methods which were published in our pervious paper in the current study.

### Senescence-associated β -galactosidase staining

hNESPCs were washed in 1 × PBS and fixed in 4% formaldehyde for 5 minutes at room temperature. Cells were then incubated overnight at 37°C with freshly prepared SA-β-gal solution (1 mg/ml X-gal, 150 mM NaCl, 2 mM MgCl_2_, 5 mM potassium ferrocyanide, 5 mM potassium ferricyanide, 40 mM sodium phosphate/citric acid at pH 6) as per the method reported by Dimri *et al.*^19^ Positive staining of senescent cells was evident from 2 h post-staining onwards, but cells were examined under light microscopy and imaged at 16 h post-staining when maximal staining occurred. During the development of staining, cells were incubated at 37°C overnight. Both of the NIH/3T3 feeder layer cells and hNESPCs were treated with 4 μg/ml of mitomycin C for 3 h and then used as a positive control for the SA-β-gal staining.

### Immunocytochemistry in cell cultures

p63 (epithelial stem cell marker) and Ki67 (proliferation marker) were stained in hNESPC cultures by immunofluorescence (IF) assay. hNESPCs to be examined by staining were grown on cover slips and were fixed at the end of the observation for doubling time (Figure 5). The cells were then incubated with primary antibodies over night at 4°C followed by 1 hr. incubation with Alexa Fluor 488-conjugated goat anti-mouse IgG (Life Technologies, Grand Island, NY) and Alexa Fluor 594-conjugated goat anti-rabbit IgG (Life Technologies) in the dark at 37°C. Rabbit anti-human p63 monoclonal antibody [Clone EPR5701] (Abcam, Cambridge, UK), mouse anti-human KRT5 monoclonal antibody [Clone XM26] (Abcam), and mouse anti-human Ki-67 monoclonal antibody [Clone Ki-S5] (Millipore, Billerica, MA) were used at dilutions of 1:200, 1:800 respectively. Species- and subtype-matched antibodies were used as negative controls [Universal negative controls for mouse or rabbit primary antibodies (Dako, Glostrup, Denmark)]. The coverslips were mounted on the slides by using SlowFade Gold antifade reagent with 4′,6-diamidino-2-phenylindole (DAPI) (Life Technologies). Images from IF slides were obtained with an inverted microscope (Olympus IX51) using ×20 objective lens. For quantifying the number of Ki67^+^ or p63^+^ cells, 3 pictures were randomly taken from each sample in a blind manner. Percentage of Ki67^+^ cells among p63^+^ cells in each clone was then calculated.

### Immunohistochemistry and immunofluorescence assays in solid tissues

p63 and Ki67 were also stained in solid tissues obtained from the same subjects whose biopsies were used for cell culture. Nasal tissues were fixed in formalin, embedded in paraffin and sectioned at 4 μm with a Leica microtome (Leica, Wetzlar, Germany). Slides were processed with Target Retrieval Buffer (Dako A/S, Glostrup, Denmark). Endogenous peroxidase activity was blocked with 3% H_2_O_2_. They were stained with the same primary antibodies of p63 and Ki67 in the same conditions described in the above section. For immunohistochemistry (IHC), the slides were incubated with DAKO EnVision^+^System-HRP (Dako A/S) at room temperature for 30 min followed by diaminobenzidine as color development. For immunofluorescence (IF), we used the same secondary Alexa Fluor antibodies (Life Technologies) as were used in the same staining conditions. Images from IHC and IF slides were obtained with an inverted microscope (Olympus IX51) using ×20 objective lens. For quantifying the number of Ki67^+^ or p63^+^ cells, two areas in the epithelium region were randomly taken from each sample in a blind manner. The percentage of Ki67^+^ cells among p63^+^ cells in each solid tissue was then calculated.

### RNA extraction and Quantitative real-time PCR

RNA was extracted from hNESPC cultures of P0, P1, and P2 with a mirVana™ miRNA Isolation Kit (Life Technologies). One microgram of total RNA was reversely transcribed to cDNA by using a Maxima Reverse Transcriptase Kit (Thermo Scientific, Rockford, IL) based on the manufacture's protocol. Real-time RT-PCR analysis was performed to evaluate the expression levels of ki67 and p63. PGK1 was used as a housekeeping control. The TaqMan assays (Life Technologies) included the following genes: p63, Hs00978343_m1; ki67, Hs01032443_m1; PGK1 Hs99999906_m1. PCR reactions were run on a Step One Plus real-time PCR machine (Life Technologies). Both target and reference (PGK1) genes were amplified in separate wells in triplicate. Relative gene expression was analyzed using the 2^−ΔΔCt^ method with a PGK1 as a reference^20^.

### Statistical analysis

Because observations of doubling time and CFE were repeated measurements per sample, Liner Mixed Models were used to estimate the difference of these two measurements between NP and controls in each passage. Mann-Whitney test was used to analyze the difference of Ki67^+^/p63^+^ cells percentage in colonies or in tissue sections between NPs and controls. The data analyses were performed by SPSS software version 18 (SPSS Inc., Chicago, IL). A *p*-value less than 0.05 indicates a statistical significance. All patients were coded confidentially, and evaluations of doubling time, colony efficiency, and immunocyto-/imunohisto-chemistry were performed independently by another researcher in a blind manner.

## Author Contributions

D.Y.W., C.W.L., X.M.Y., X.L.P. and L.S. designed the study. S.S.C. provided the clinical biopsies. X.M.Y., C.W.L., S.S.C., Y.Y.L., Y.Y., X.N.Z., F.G.Y. and J.L. collected and processed the clinical biopsies. X.M.Y., Y.Y.L., X.N.Z., F.G.Y., J.L. and Y.Y. did the cell culture experiment. X.M.Y., C.W.L., Y.Y. and Y.Y.L. did the staining and PCR experiments. C.W.L., X.M.Y., L.S., D.Y.W., X.L.P. and L.S. did the data analysis. C.W.L., X.M.Y., D.Y.W., X.L.P. and L.S. wrote the main manuscript. All authors reviewed the manuscript.

## Supplementary Material

Supplementary Information Supplementary Information File
