# Supplementary material for: Reduced growth and proliferation dynamics of nasal epithelial stem/progenitor cells in nasal polyps in vitro
Source: Sci Rep. 2014 Apr 9;4:4619. doi: 10.1038/srep04619 (PMC3980221; doi:10.1038/srep04619)
Supplement: Supplementary Information — File [file srep04619-s1.pdf]

Supplementary information

Reduced growth and proliferation dynamics of nasal epithelial stem/progenitor cells in nasal polyps *in vitro*

Xue Min Yu [1,2†], Chu Wei Li [2†], Siew Shuen Chao [2], Ying Ying Li [2], Yan Yan [2], Xue Ning Zhao [1,3], Feng Gang Yu [2], Jing Liu [2], Liang Shen [4], Xin Liang Pan [1], Li Shi [1*], De Yun Wang [2*]

[1] Department of Otolaryngology, Qilu Hospital, Shandong University, China

[2] Department of Otolaryngology, National University of Singapore, National University Health System, Singapore

[3] Department of General Health Care Ⅱ, Shandong Provincial Hospital affiliated to Shandong University, China

[4] Biostatistics Unit, National University of Singapore, National University Health System, Singapore

[†]: Yu XM and Li CW contributed equally to this work.

[*] **Corresponding authors**

De Yun Wang, MD, PhD

Department of Otolaryngology

National University of Singapore

National University Health System

1E Kent Ridge Road

Singapore 119228

Phone: +65 67725373

Fax: +65 67753820

E-mail: entwdy@nus.edu.sg

Li Shi, MD, PhD

Department of Otolaryngology, Qilu Hospital, Shandong University

44 Wenhua West Road, Jinan, Shandong, P. R. China, 250012

Phone: +86 531 82169216

Fax: +86 531 87332617

Email: shili126@sina.com

Table S1 Correlation between age and measurements of cell proliferation

Subject	Diagnosis	Sex	Age	CFE (%)				Doubling time			
				P0	P1	P2	P3	P0	P1	P2	P3
1	NP	M	54	10.5	22.1	13.4	3.8	33.1	19.8	32.3	76.1
2	NP	M	50	1.7	20.1	6.4	0.6	33.8	17.9	49.9	44.6
3	NP	M	46	0.7	6.2	5.6	*NA*	53.0	38.7	27.0	*NA*
4	NP	M	50	5.9	7.1	3.0	*NA*	36.5	22.3	42.8	*NA*
5	Control	M	25	7.3	35.3	18.8	12.9	16.9	13.3	16.4	22.1
6	Control	F	45	8.9	24.8	12.9	3.9	30.3	12.7	28.1	16.9
7	Control	M	42	21.7	28.6	18.7	12.5	14.4	15.2	19.7	21.6
8	Control	M	50	4.1	27.2	19.4	14.5	18.5	16.1	20.5	26.8
Correlation with Age*				*NS*	*NS*	*NS*	*NS*	*NS*	*NS*	*NS*	*NS*

* Relationship between age and data of CFE/doubling time in each passage were analyzed by Pearson correlation method. A p-value less than 0.05 was considered significant. *NS*, non-significant. *NA*, not applicable.

Figure Legends

Figure S1 Representative pictures for staining of KRT14 in both cell cultures and tissues. KRT14 was double stained with p63 (A) and Ki67 (B) in the colonies. Double staining of KRT14 with p63 in tissue sections from healthy control (C) and while only in the tissue sections from NP patients (D). Original magnification for pictures A & B at 200× and for pictures C, D at 200×, respectively (scale bar =50 μm).

Figure S2 Representative pictures showed the cells with "fried-egg" shape (A) and showed the β-galactosidase (β-gal) staining in panel (B): a colony of P1 cells (a), in a colony of mitomycin C-induced P1 cells as a positive control for the staining (b), in the colonies of P3 cells from NP samples (c & d), in the colonies of P3 cells from a control sample (e). All the β-gal staining photos were captured at 200× magnification.

(scale bar =50 μm).

Figure S3 Comparison of $Ki67^{+}/p63^{+}$ cell percentage between NP and control tissues. Statistical analyses were performed with Mann-Whitney test. The data are present as percentage (median, 25th-75th percentile) in the plot.

Figure S4 Representative pictures for observation of colony-forming efficiency (A-B) and doubling time (C-E). More colonies were found in cultures from healthy controls (A) as compared to those from patients with nasal polyposis (B). "*" represents the colonies which matched our criteria to be considered a "positive" colony. Original magnification for pictures A and B, 100× (scale bar =100 μm).. Pictures C, D and E were taken for the same colony at different time points (Day1, Day2, Day3), which were used for evaluation of doubling time. Original magnification for pictures C, D, and E, 200×(scale bar =50 μm).

Figure S5 Cell proliferation assay using CyQUANT® kit was performed in hNESPCs from NP and control samples during passage 0 (P0) to P3. Each data point was presented as mean ± SE.

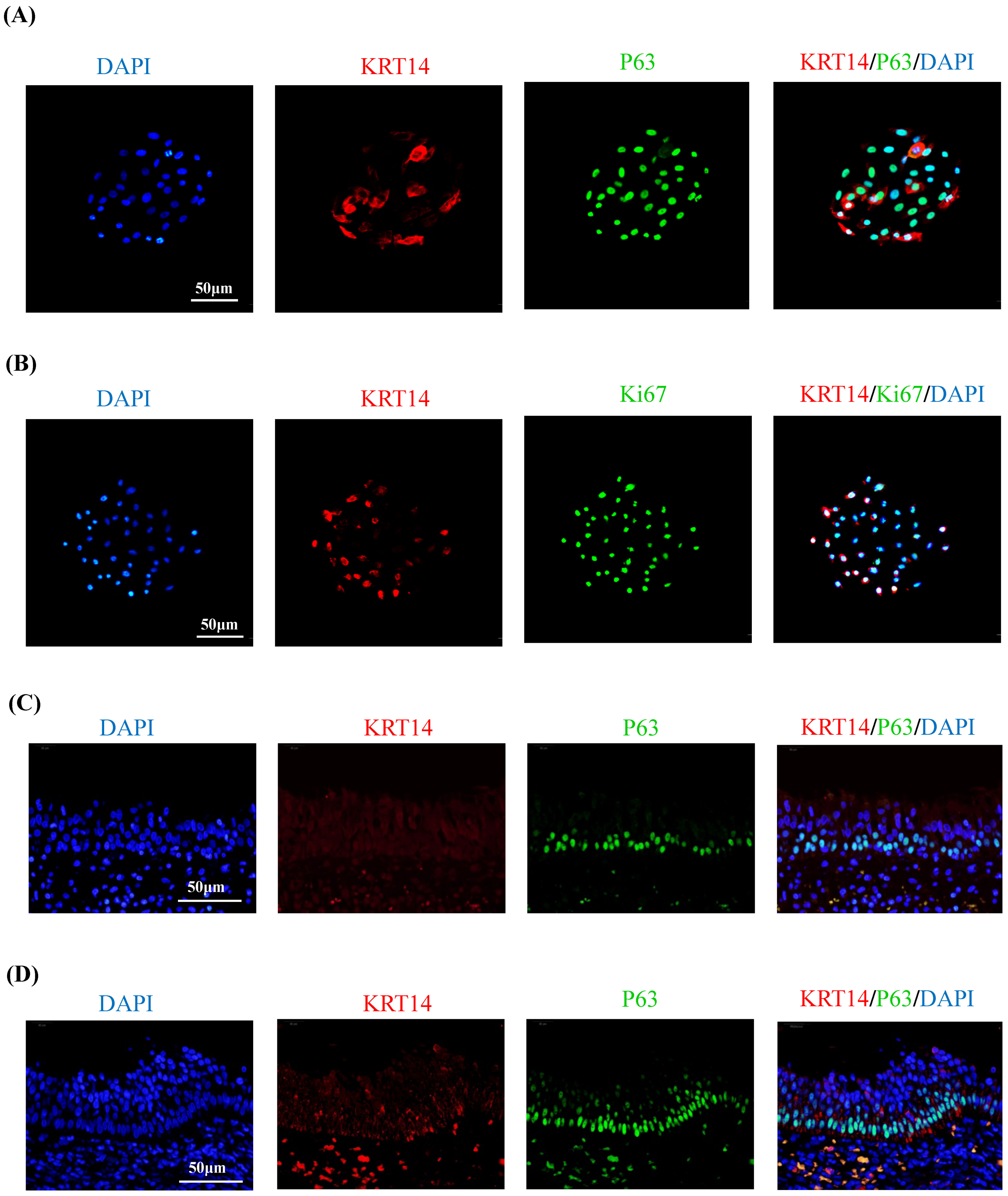
(A)
DAPI
KRT14
P63
KRT14/P63/DAPI
50μm
(B)
DAPI
KRT14
Ki67
KRT14/Ki67/DAPI
50μm
(C)
DAPI
KRT14
P63
KRT14/P63/DAPI
50μm
(D)
DAPI
KRT14
P63
KRT14/P63/DAPI
50μm

(A)

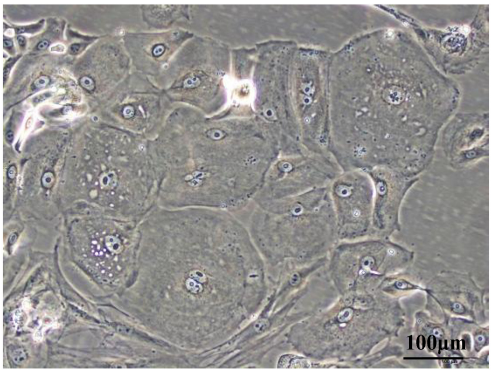
100µm

(B)

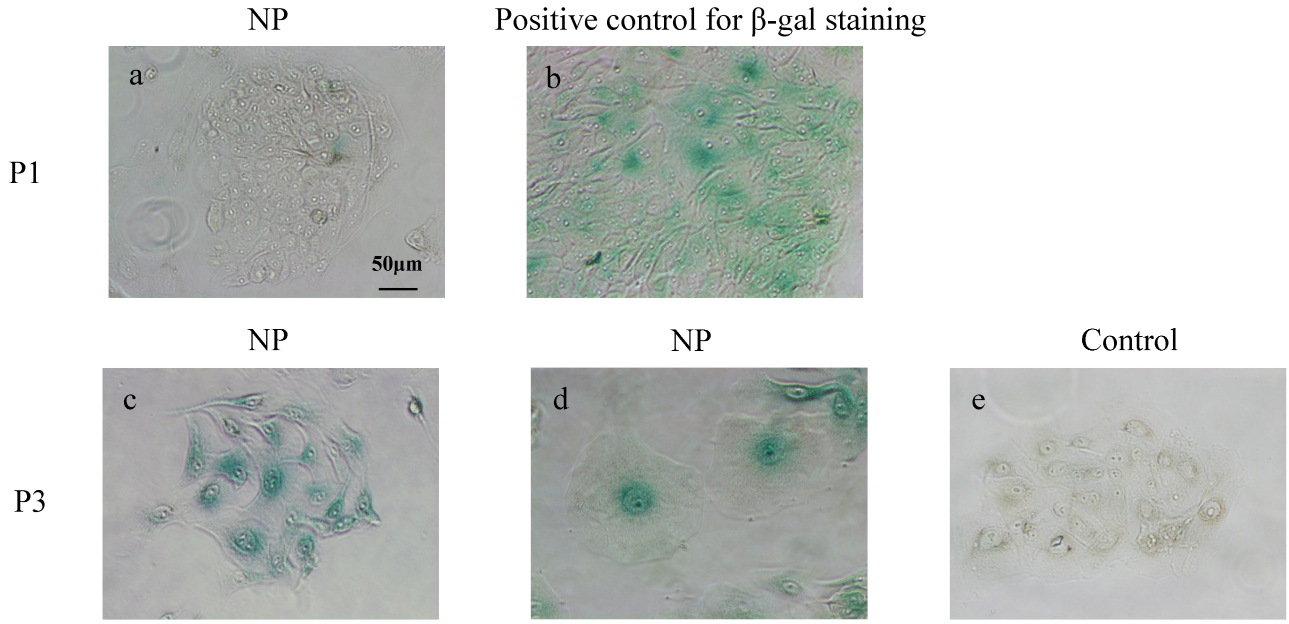
NP
Positive control for β-gal staining
P1
a
50µm
b
NP
NP
Control
P3
c
d
e

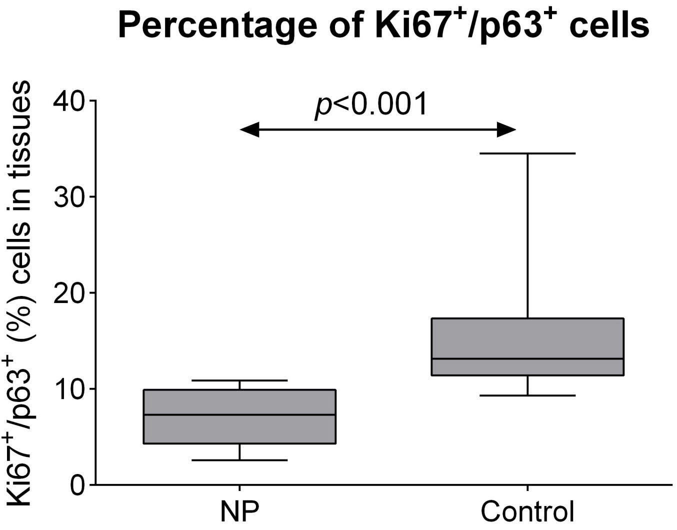

Percentage of Ki67+/p63+ cells
Ki67+/p63+ (%) cells in tissues
40
30
20
10
0
p<0.001
NP
Control

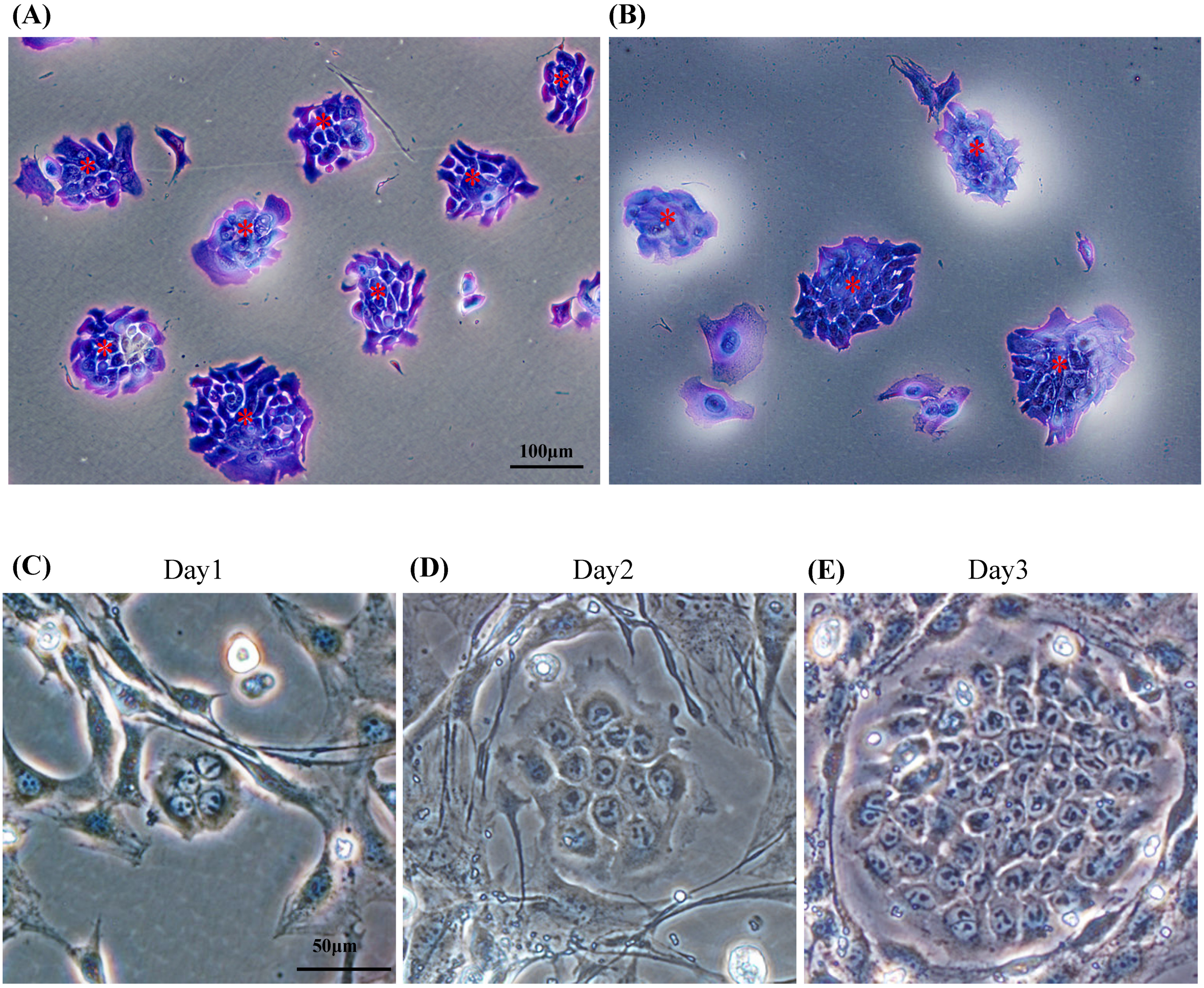

(A)
(B)
100μm
(C)
Day1
(D)
Day2
(E)
Day3
50μm

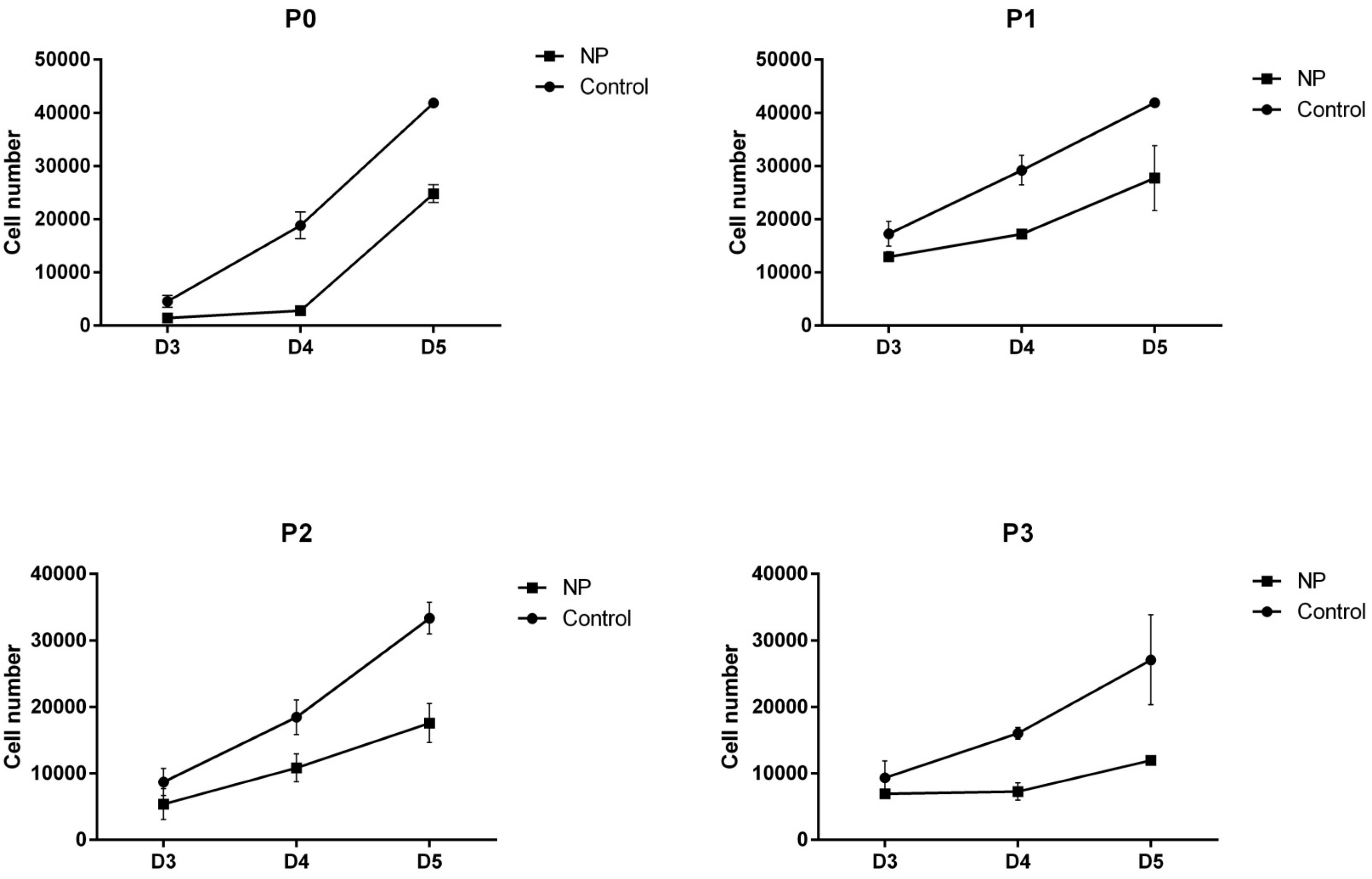
P0
NP
Control
Cell number
50000
40000
30000
20000
10000
0
D3
D4
D5
P1
NP
Control
Cell number
50000
40000
30000
20000
10000
0
D3
D4
D5
P2
NP
Control
Cell number
40000
30000
20000
10000
0
D3
D4
D5
P3
NP
Control
Cell number
40000
30000
20000
10000
0
D3
D4
D5
